# Supplementary material for: Transmembrane domains of fusion proteins promote stalk formation by inducing membrane disorder
Source: Biophys J. 2026 Jan 21;125(5):1276–85. doi: 10.1016/j.bpj.2026.01.032 (PMC13351912; doi:10.1016/j.bpj.2026.01.032)
Supplement: Document S1. Figures S1–S16 and Tables S1–S5 [file mmc1.pdf]

Biophysical Journal, Volume *125*

Supplemental information

Transmembrane domains of fusion proteins promote stalk formation by inducing membrane disorder

Katharina C. Scherer, Chetan S. Poojari, and Jochen S. Hub

Supplementary information for: Transmembrane domains of fusion proteins promote stalk formation by inducing membrane disorder

Katharina C. Scherer, Chetan S. Poojari, and Jochen S. Hub*

Theoretical Physics and Center for Biophysics, Saarland University, Saarbrücken, Germany

E-mail: jochen.hub@uni-saarland.de

Phone: +49 (0)681 302-2740. Fax: +49 (0)681 302-2748

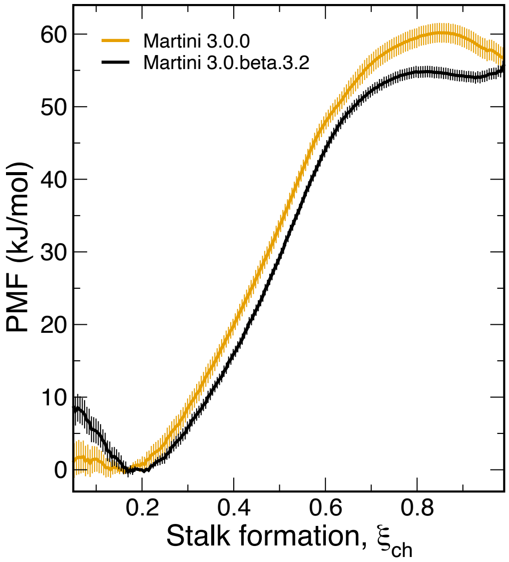

Supplementary Figure S1: Comparison of PMFs of stalk formation between membranes of pure POPC obtained with the two types of simulation setups used in this study. Black: Simulation carried out with Martini 3.0.beta.3.2 and system build with 128 lipids per bilayer, corresponding to an area of $\sim 40\,\mathrm{nm}^2$, and using 8 water beads per nm^2 between bilayers. Orange: Carried out with Martini 3.0.0 and system build with a membrane area of $100\,\mathrm{nm}^2$ and using 12 water beads per nm^2 between the bilayers. For the Martini 3.0.0 setup, we chose the number of water beads between the bilayers such that the PMF agrees approximately with the PMF obtained with the Martini 3.0.beta.3.2 system.

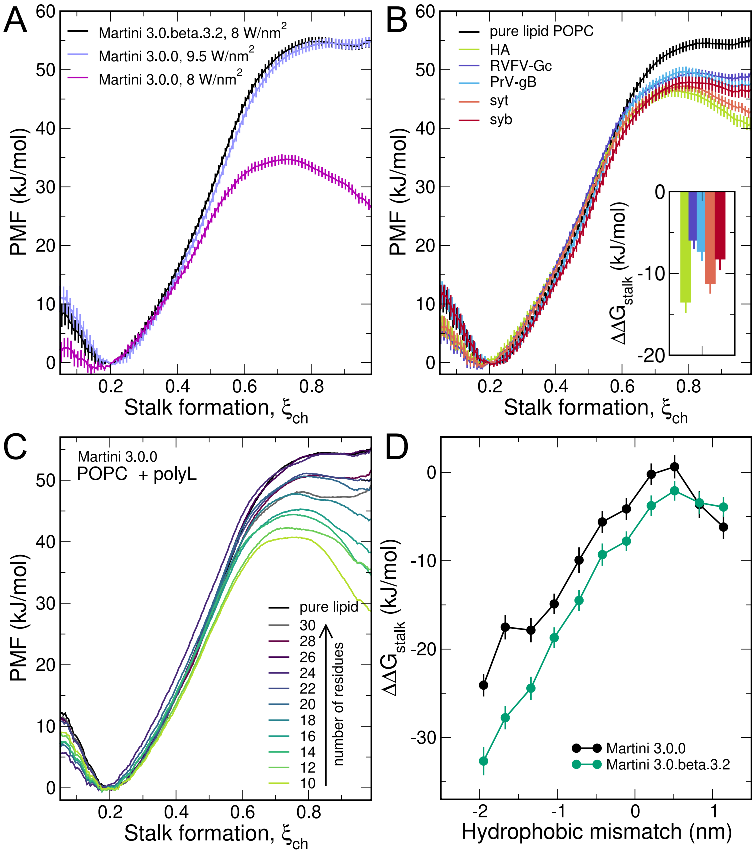

Supplementary Figure S2: Additional control simulations of comparing simulations using Martini version 3.0.beta.3.2 (v3beta) or with simulations using Martini 3.0.0 (v3). The results highlight that quantitative comparison of stalk free energies obtained with v3 and v3beta requires care. However, since the trends are conserved, differences among v3 and v3beta do not affect the conclusions of this study. (A) PMFs of stalk formation between POPC bilayers with 128 lipids per bilayer and 8 waters per nm^2 with v3beta (black) or v3 (pink), revealing that the v3 membranes is more fusogenic than the v3beta membrane given the same number of water molecules per area in the proximal compartment. This discrepancy arises from different headgroup–headgroup distances between the proximal leaflets: 8 waters per nm^2 yield distances of 1.45 nm and 1.63 nm with v3 and v3beta, respectively. Headgroup–headgroup distance strongly influences the stalk free energy[1] (see Fig. S15). Upon matching the headgroup–headgroup distance by simulating v3 with 9.5 instead of 8 waters per nm^2, the PMFs agree (black and purple). (B) PMFs of stalk formation between POPC bilayers with one TMD from influenza virus hemagglutinin (HA, green), Rift Valley fever virus Gc (RVFV-Gc, purple), pseudorabies virus glycoprotein B (PrV-gB, blue), sytaxin (syt, orange), or synaptobrevin (syb, red) with v3 and 9.5 waters per nm^2. Comparison with Figure 3A and S10 demonstrate that the effect of the TMDs on $\Delta\Delta G_{stalk}$ agree among v3 and v3beta. (C) PMFs of stalk formation between v3 POPC bilayers with one polyL helix each ($R_2L_nR_2$, $n = 6, 8, 10, ..., 26$) each with increasing hydrophobic length and 9.5 waters per nm^2, thereby using the same headgroup distance as with v3beta (compare with Fig. S8, and see panel A). (D) Change in stalk free energy, $\Delta\Delta G_{stalk}$, versus hydrophobic mismatch between polyleucine helices and the membrane core for v3 (black) and v3beta (cyan). Trends in stalk stabilization by TMDs are reasonably consistent among v3 and v3beta.

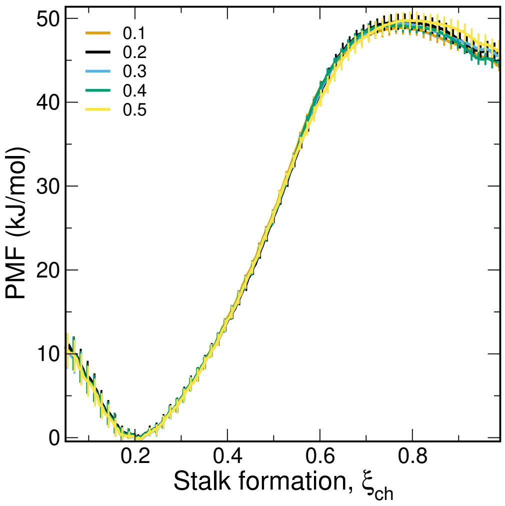

Supplementary Figure S3: Additional control simulations on the use of flat-bottomed restraints for keeping the TMD center of mass near the membrane center. PMFs of stalk formation between two POPC bilayers with one TMD from Rift Valley fever virus Gc and varied thickness of the flat region in the flat-bottomed restraints applied on the central TMD backbone bead. Modifying the flat-bottom thickness radius between 0.1 nm to 0.5 nm has no effect on the PMFs.

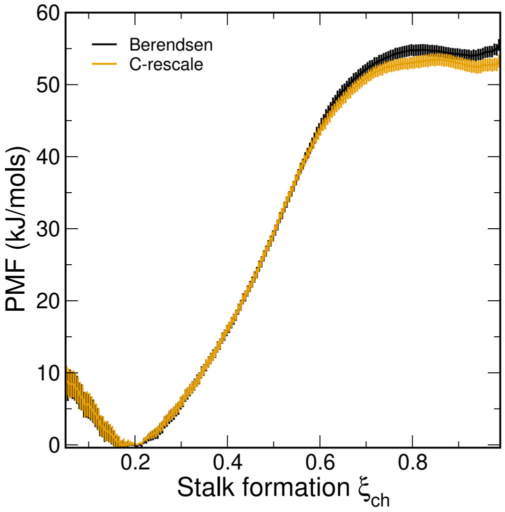

Supplementary Figure S4: PMFs of stalk formation between two POPC bilayers with two different pressure coupling algorithms: Berendsen (black) and C-rescale (orange). The PMFs agree within statistical errors, indicating that the choice of barostat has only a marginal effect (if any).

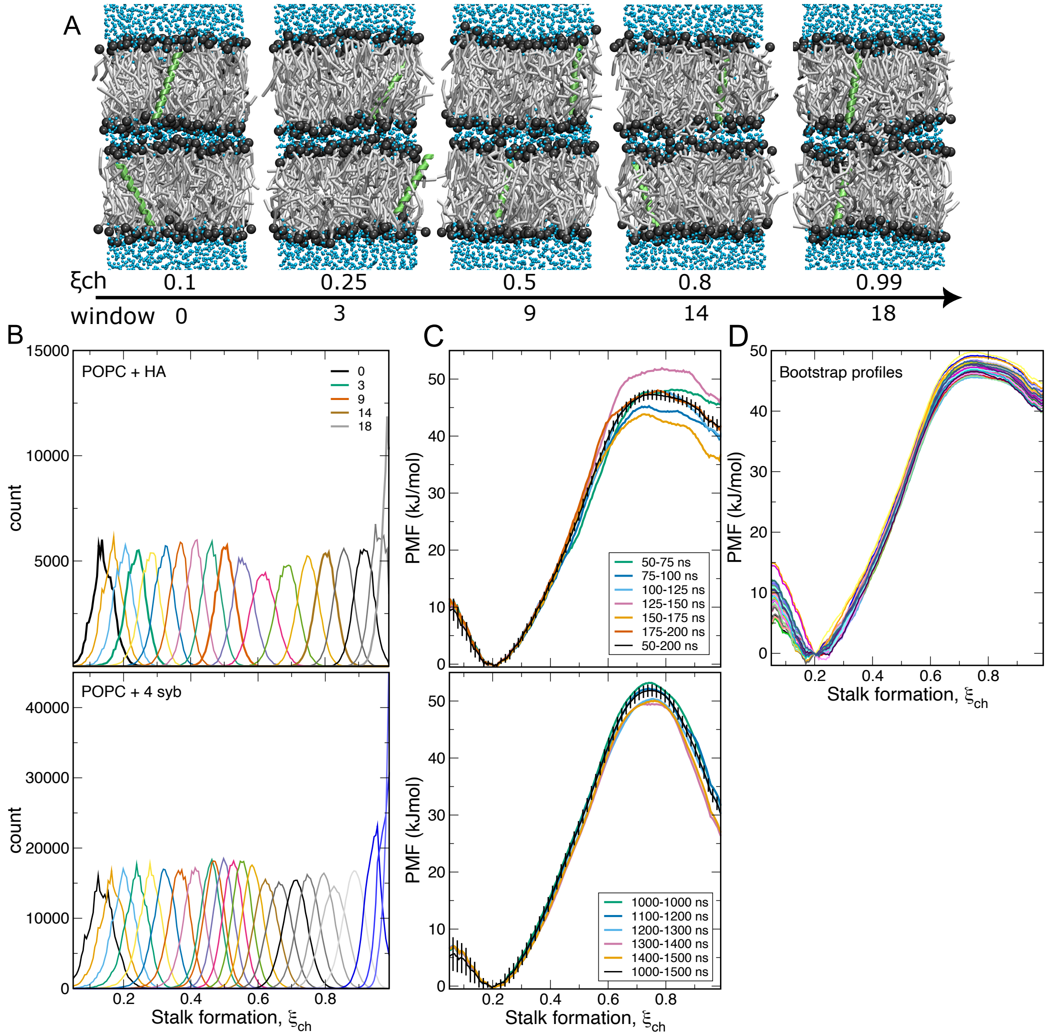

Supplementary Figure S5: Analysis of convergence of PMF calculations. (A) Simulation snapshots of POPC bilayers with TMD from influenza hemagglutinin (HA) of umbrella windows along ξ_{ch}. Histograms from these umbrella windows are highlighted in panel B (top). (B) Umbrella histograms and block-averaged PMFs for a Martini 3.0.beta.3.2 simulation system with one TMD per bilayer (top) and Martini 3.0.0 with 4 TMD per bilayer (bottom). Histograms show sufficient overlap along ξ_{ch}. Note that non-Gaussian shapes of the the histograms do not indicate poor convergence but stems from discrete, step-wise definition of connectivity by the chain reaction coordinate ξ_{ch}. (C) PMFs obtained from the histograms in panel B from time blocks (see legend), demonstrating that the (i) PMFs are converged and not affected by long autocorrelation times, and that (ii) the errors estimated by bootstrapping of histograms (black error bars, denoting 1 SE) are reasonable. (D) Bootstraped profiles used for error estimation as used for obtaining error bars shown in panel B (black PMF). The uncertainties were estimated using 50 rounds of Bayesian bootstrapping of complete histograms, as implemented by gmx wham.[2] Accordingly, in each round of bootstrapping random weights are assigned to the histograms and a new synthetic, bootstrapped PMF is obtained. The standard error is obtained from the standard deviation among the bootstrapped PMFs. While errors have been computed for all PMFs in this study, they are usually not shown for clarity. They have, however, been used to obtain the error of the $\Delta\Delta G_{\mathrm{stalk}}$ values (see Tables S3–S5). Error bars computed by bootstrapping denote 1 SE.

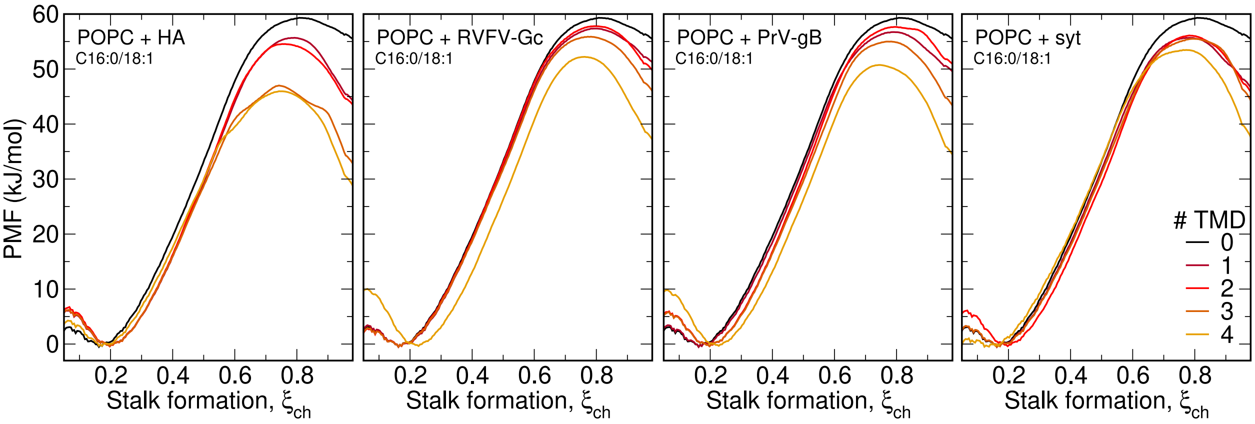

Supplementary Figure S6: PMFs of stalk formation for POPC bilayers with zero to four TMDs (for color code, see legend) from the following fusion proteins (from left to right): influenza virus hemagglutinin (HA), Rift Valley fever virus Gc (RVFV-Gc), pseudorabies virus glycoprotein B (PrV-gB), syntaxin (syt).

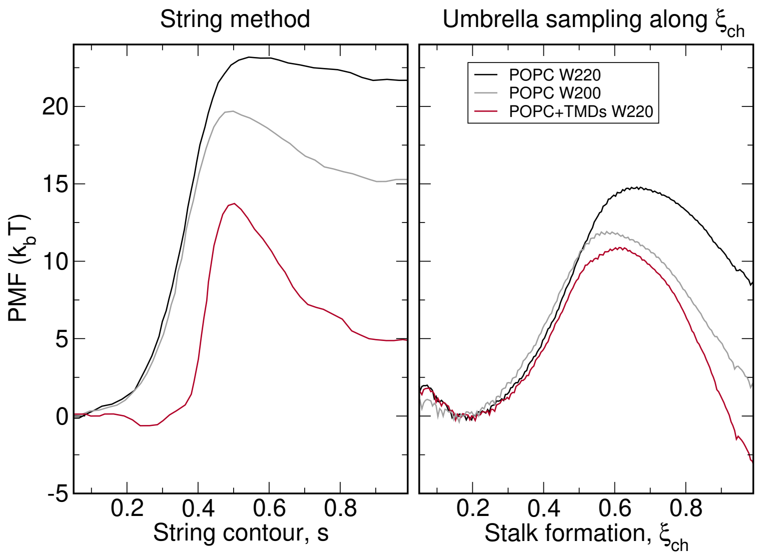

Supplementary Figure S7: Comparison of PMFs of stalk formation along the chain coordinate ξ_{ch} with previous work by Smirnova *et al.*[3] Left: Minimum free-energy path for stalk formation between two POPC bilayers at two different degrees of hydration (W220, W200) and with inserted TMDs of the SNARE complex for the W200 system, taken from Ref. 3.
Right: PMFs computed for the same simulation systems, kindly provided by Smirnova *et al.*,[3] however obtained with umbrella sampling along ξ_{ch}.
Effects of different degrees of hydration and the effect of the TMD agree qualitatively between the two methods. However, PMFs computed along ξ_{ch} suggest smaller free energies of stalk formation as compared to Ref. 3. This difference may be rationalized by the different definitions of the stalk states. By $\xi_{\mathrm{ch}} \approx 1$, the stalk state is defined by the presence of a hydrophobic connection between the two membranes, whereas the stalk shape, thickness, and lateral position of the connection are freely chosen and sampled by the force field. In Ref. 3, the stalk state is defined with a specific 3D density, which might allow fewer conformational states compared to the $\xi_{\mathrm{ch}} \approx 1$ stalk definition, thereby possibly leading to lower entropy and higher free energy.

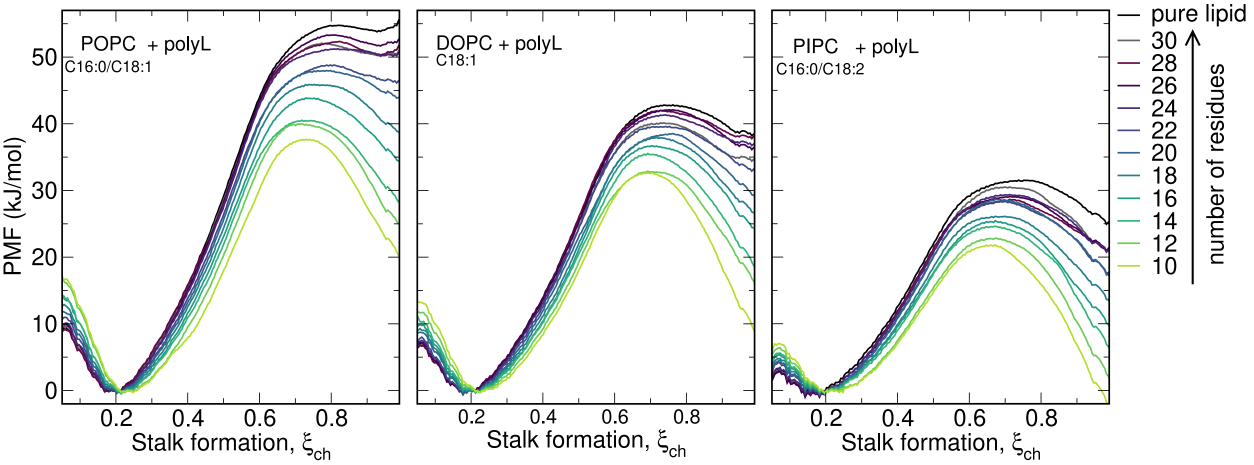

Supplementary Figure S8: PMFs of stalk formation between membranes of (from left to right) POPC, DOPC, or PIPC with one polyleucine helix (polyL) each with increasing hydrophobic length as controlled by the sequence $R_2L_nR_2$ ($n = 6, 8, 10, ..., 26$). The black line shows the PMF for pure lipid bilayers for reference.

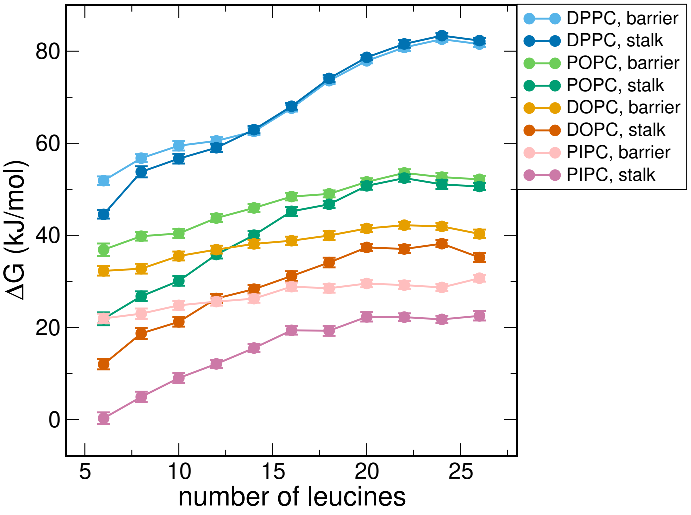

Supplementary Figure S9: Free energy of stalk formation and free energy barrier versus number of leucines in polyL helices $R_2L_nR_2$ ($n = 6, 8, 10, ..., 26$), defined as the PMF average for $\xi_{ch} > 0.96$ or as the PMF maximum between $\xi_{ch} > 0.3$ and $\xi_{ch} < 0.953$, respectively. PMFs are shown in Figs. S8 and 2B. Free energy of stalk formation and the free energy barrier increase with the number of leucines in polyL.

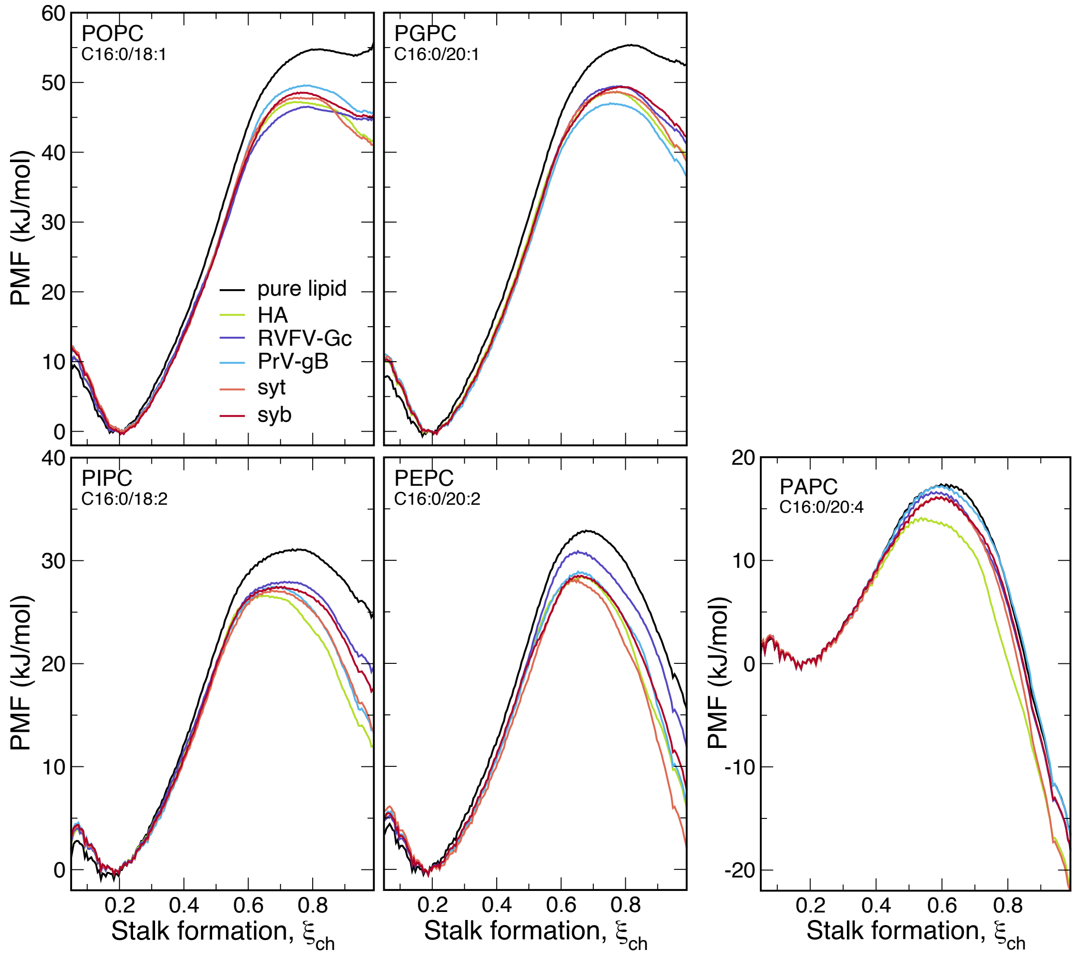

Supplementary Figure S10: PMFs of stalk formation between membranes composed of POPC, PGPC, PIPC, PEPC or PAPC (see labels) with one TMD from influenza virus hemagglutinin (HA, green), Rift Valley fever virus Gc (RVFV-Gc, purple), pseudorabies virus glycoprotein B (PrV-gB, blue), sytaxin (syt, orange), or synaptobrevin (syb, red). The black line shows the PMF for pure lipid bilayers for reference.

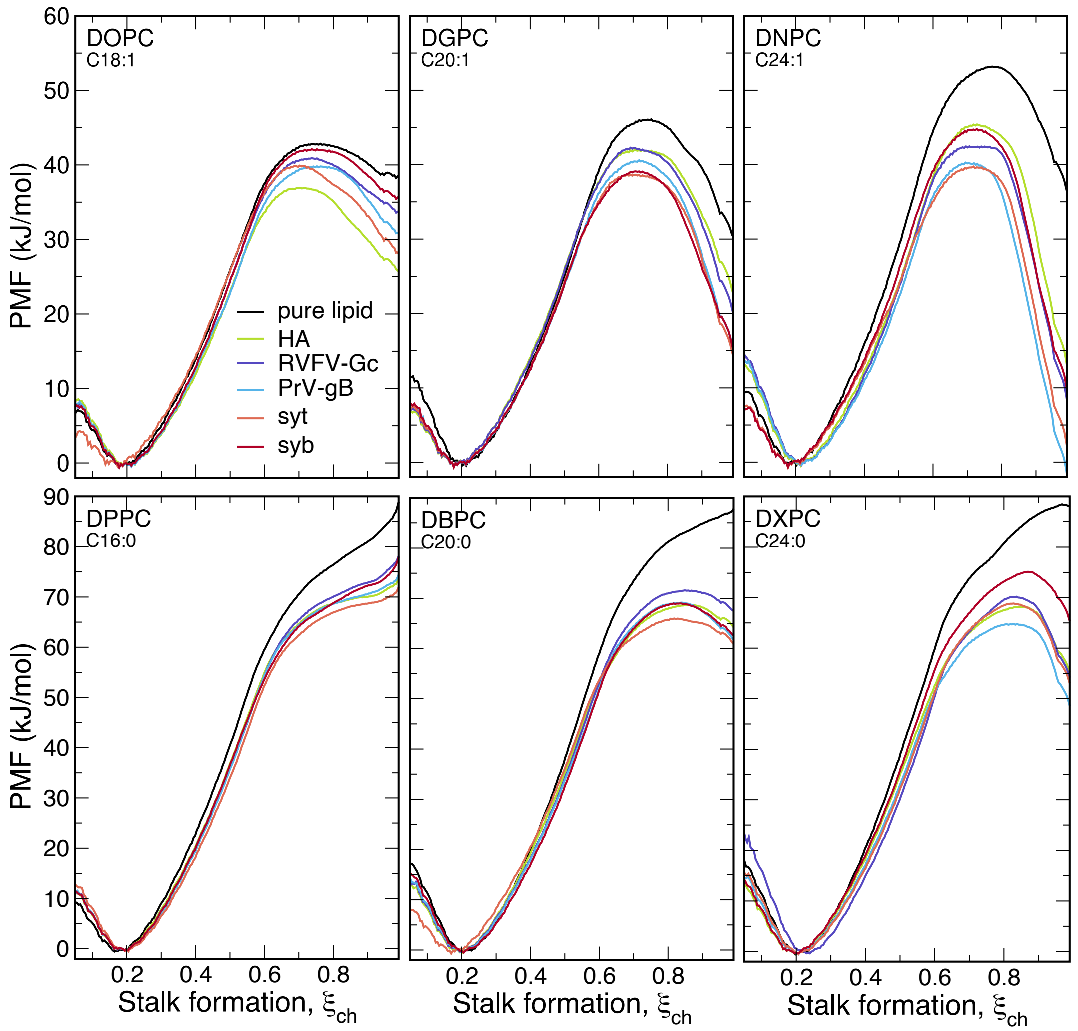

Supplementary Figure S11: PMFs of stalk formation between membranes composed of DOPC, DGPC, DNPC, DPPC, DBPC, or DXPC with one TMD from influenza virus hemagglutinin (HA, green), Rift Valley fever virus Gc (RVFV-Gc, purple), pseudorabies virus glycoprotein B (PrV-gB, blue), sytaxin (syt, orange), or synaptobrevin (syb, red). The black line shows the PMF for pure lipid bilayers for reference.

Lipid	Thickness (nm)
DBPC	3.57
DGPC	3.25
DNPC	3.90
DOPC	2.60
DPPC	2.85
DXPC	4.25
PAPC	2.66
PEPC	2.86
PGPC	3.02
PIPC	2.57
POPC	2.69

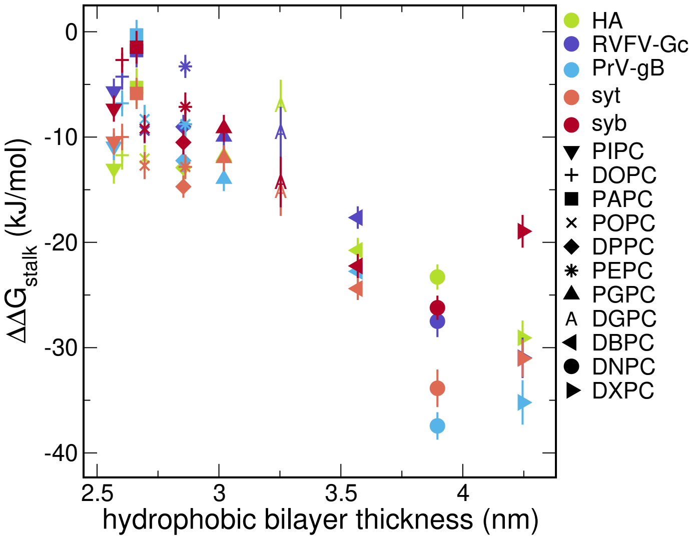

Supplementary Figure S12: (*Left*) Hydrophobic core thickness of pure lipid bilayers. (*Right*) Change in stalk free energy $\Delta\Delta G_{stalk}$ upon insertion of one TMD from influenza virus hemagglutinin (HA), Rift Valley fever virus Gc (RVFV-Gc), pseudorabies virus glycoprotein B (PrV-gB), sytaxin (syt), or synaptobrevin (syb) (see colors) versus hydrophobic thickness of membranes composed of PIPC, DOPC, PAPC, POPC, DPPC, PEPC, PGPC, DGPC, DBPC, DNPC or DXPC (see symbols). The stalk-stabilizing effect by the TMDs is increased with increased bilayer thickness.

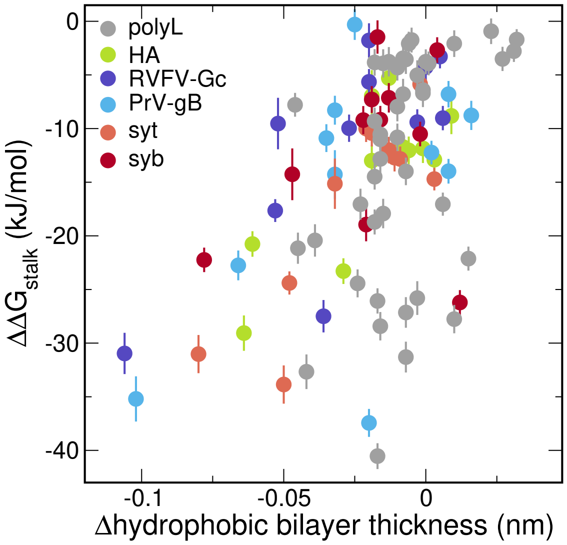

Supplementary Figure S13: Change in stalk free energy $\Delta\Delta G_{stalk}$ versus change in hydrophobic bilayer thickness upon insertion of one TMD per bilayer. The correlation between $\Delta\Delta G_{stalk}$ and the change in hydrophobic bilayer thickness is weaker than its correlation with tail order (Figure 3C).

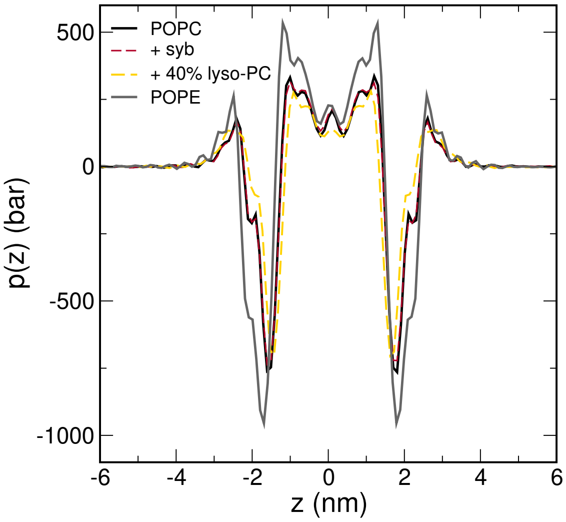

Lipids	Bending moment (bar·nm^2)
POPC	-212 ± 4
+RVFV-Gc	-212 ± 8
+PrV-gB	-210 ± 4
+syt	-212 ± 6
+syb	-217 ± 6
+10% lyso-PC	-193 ± 6
+20% lyso-PC	-174 ± 5
+40% lyso-PC	-97 ± 6
POPE	-371 ± 4

Supplementary Figure S14: TMDs do not facilitate stalk formation by modulating membrane bending. (*Left*) Lateral pressure profiles form single-bilayer simulations of pure POPC, POPC with one TMD of synaptobrevin, mixture of 60% POPC and 40% lysophosphatidylcholine (lyso-PC) and pure POPE (see legend) obtained with Martini 3.0.beta.3.2. (*Right*) Bending moments with 1 SE of bilayers of pure POPC, POPC plus one TMD, POPC plus lyso-PC (10%, 20%, or 40%), or POPE. Evidently, bending moments do not change within errors upon TMD insertion. In contrast, replacing 10% to 40% of POPC with lyso-PC or replacing POPC with POPE greatly changes the bending moment, as expected owing to the inverted-cone and cone shapes of lyso-PC and POPE, respectively. Notably, membranes of POPE or of POPC/lyso-PC 90:10 reveal stalk free energies that are decreased or increased by ∼15 kJ/mol relative to pure POPC,[1] in line with the expectation that negative and positive intrinsic curvatures favor and disfavor stalk formation. These findings (i) are compatible with the notion that that POPE and lyso-PC modulate stalk formation by modulating membrane bending energies, but (ii) that TMDs favor stalk formation by a different mechanism. Bending moments were obtained from lateral pressure profiles $p(z)$ from single-bilayer simulations. The bending moment is defined by the first moment of the pressure profile: $\kappa C_0 = \int_0^l z p(z) \mathrm{d}z$, where κ is the bending modulus and C_0 the spontaneous curvature. The profiles $p(z)$ were computed with Gromacs-LS.[4,5] Integration was performed separately for each monolayer, followed by averaging. The reported values are the averages over two monolayer and were obtained from 3 μs simulation, with coordinates and velocities saved every 30 ps. Integration was done in 10 blocks with 10.000 frames each.

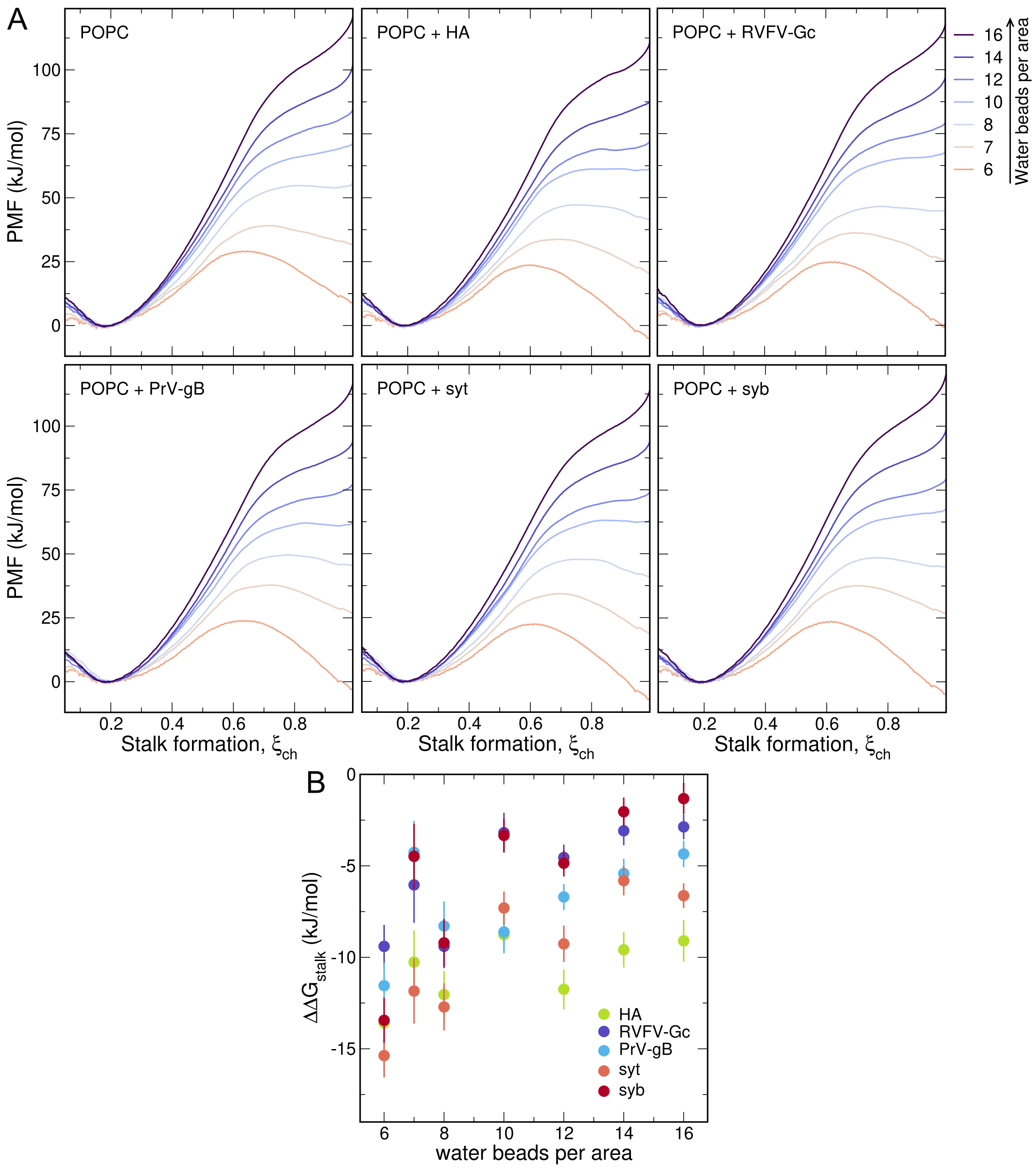

Supplementary Figure S15: Additional controls simulations using different degrees of hydration betweeen the proximal leaflets as controlled by the number of water beads per nm^2. (A) PMFs of stalk formation between membranes of POPC with one TMD from influenza virus hemagglutinin (HA), Rift Valley fever virus Gc (RVFV-Gc), pseudorabies virus glycoprotein B (PrV-gB), sytaxin (syt), or synaptobrevin (syb) (see lables) and varied hydration levels from 4 to 16 waters/nm^2 (see legend). (B) Change in stalk free energy $\Delta\Delta G_{stalk}$ upon insertion of one TMD versus water beads per area. While the hydration level greatly influences the absolute value of the stalk free energy (PMFs in panel A), the relative change in stalk free energy upon insertion of one TMD, $\Delta\Delta G_{stalk}$, shows no systematic dependence on bilayer hydration and remains similar across a broad range of hydration levels.

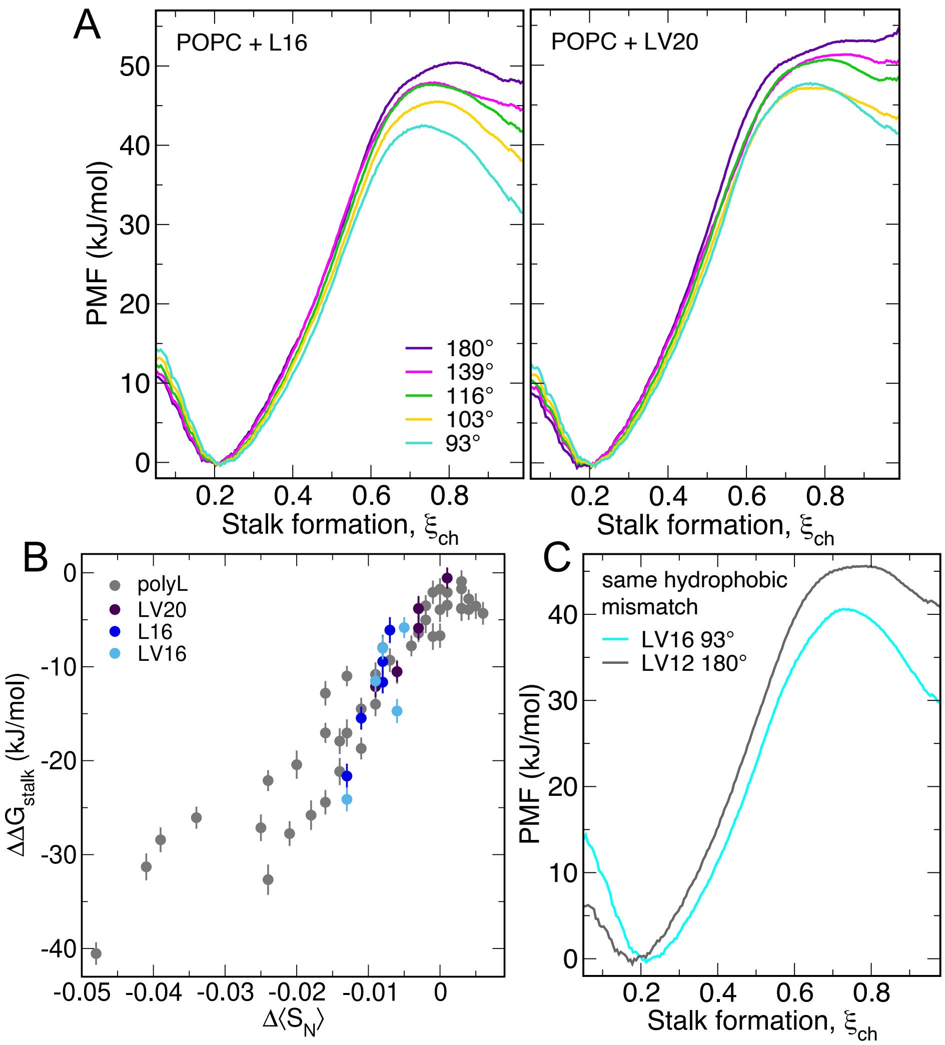

Supplementary Figure S16: (A) PMFs of stalk formation between two POPC bilayers with inserted L16 ($K_3W(LV)_{16}K_3$, left) or LV20 ($K_3W(LV)_{10}K_3$, right) with different bending angles. (B) Change in stalk free energy $\Delta\Delta G_{stalk}$ versus change in order parameter $\Delta\langle S_N\rangle$ upon insertion of one LV20 TMD ($K_3W(LV)_{10}K_3$, dark violet), one L16 TMD ($K_3WL_{16}K_3$, blue), or one LV16 TMD ($K_3W(LV)_8K_3$, light blue) per bilayer with varied bending angles. For reference, values obtained for polyleucine TMDs are shown as gray dots. (C) PMFs of stalk formation between two POPC membranes with kinked LV16 TMDs ($K_3W(LV)_8K_3$) or with straight LV12 TMD ($K_3W(LV)_6K_3$). The two TMDs yield the same hydrophobic mismatch, while the kinked LV16 TMD decreases the stalk free energy more compared to the straight LV12 TMD, demonstrating that kinks in TMDs favor stalk formation additionally to effects by negative hydrophobic mismatch.

Supplementary Table S1: Lipid naming according Martini nomenclature, full name and atomistic equivalent.

Martini	full name	atomistic
DBPC	Diarachidoylphosphatidylcholine	di–C20:0–C22:0 PC
DGPC	Di-gondoic-acid-phosphatidylcholine	di–C20:1–C22:1 PC
DNPC	Di-nervonic-acid-phosphatidylcholine	di–C24:1–C26:1 PC
DOPC	Dioleoylphosphatidylcholine	di–C16:1–C18:1 PC
DPPC	Dipalmitoylphosphatidylcholine	di–C16:0–C18:0 PC
DXPC	Dilignoceroylphosphatidylcholine	di–C24:0–C26:0 PC
PAPC	1-stearoyl-2-arachidonoyl-phosphatidylcholine	C16:0/20:4 PC
PEPC	1-stearoyl-2-eicosadienoyl-phosphatidylcholine	C16:0/20:2 PC
PGPC	1-palmitoyl-2-docosenoyl-phosphatidylcholine	C16:0/20:1 PC
PIPC	1-palmitoyl-2-linoleoyl-phosphatidylcholine	C16:0/18:2 PC
POPC	1-palmitoyl-2-oleoyl-phosphatidylcholine	C16:0/18:1 PC

Supplementary Table S2: Amino acid (aa) sequences of transmembrane domains (TMD).

fusion protein	aa sequence of TMD
influenza hemagglutinin (HA)	WILWISFAISCFLLCVVLLGFIM
Rift Valley fever virus Gc (RVFV-Gc)	TILLICLYVALSIGLFFLLIYLG
pseudorabies Virus gB (PrV-gB)	NPFGALAIGLLVLAGLVAAFLAY
syntaxin (syt)	KIMIIICCVILGIIIASTIGGIFG
synaptobrevin (syb)	MMIILGVICAIILIIIIVYFST
polyleucine (polyL)	$R_2L_nR_2$, $n = 6, \ldots, 26$
L16	$K_3W(L)_{16}K_3$
LV12	$K_3W(LV)_6K_3$
LV16	$K_3W(LV)_8K_3$
LV20	$K_3W(LV)_{10}K_3$

Supplementary Table S3: Stalk free energies in kJ/mol with error from bootstrapping (1 SE) for lipid bilayers with one TMD extracted from PMFs shown in Supplementary Fig. S10 and S11

Lipid	pure	HA	RFVF-Gc	PrV-gB	syt	syb
POPC	54.5 ± 0.8	42.5 ± 1.0	45.1 ± 0.9	46.2 ± 1.1	41.8 ± 1.0	45.3 ± 1.0
PGPC	53.0 ± 0.9	41.1 ± 1.0	43.0 ± 0.9	39.0 ± 0.8	41.0 ± 0.9	43.8 ± 0.9
PIPC	26.0 ± 0.9	13.0 ± 1.1	20.4 ± 0.8	15.2 ± 0.9	15.6 ± 0.8	18.7 ± 0.9
PEPC	18.0 ± 0.8	9.2 ± 1.5	14.7 ± 0.8	9.2 ± 1.1	5.2 ± 0.9	10.9 ± 1.1
PAPC	-12.8 ± 1.0	-18.1 ± 1.5	-14.6 ± 1.2	-13.1 ± 1.0	-18.6 ± 1.1	-14.2 ± 1.2
DOPC	39.1 ± 0.9	27.4 ± 1.0	34.8 ± 0.8	32.3 ± 0.8	29.1 ± 0.9	36.4 ± 0.8
DGPC	33.6 ± 2.1	26.7 ± 1.1	24.1 ± 1.1	19.4 ± 0.7	18.5 ± 0.9	19.4 ± 1.1
DNPC	40.6 ± 0.8	17.3 ± 0.9	13.1 ± 1.3	3.1 ± 1.1	6.7 ± 1.6	14.4 ± 0.9
DPPC	85.1 ± 0.8	72.2 ± 0.8	76.1 ± 0.8	72.8 ± 0.8	70.4 ± 0.7	74.6 ± 0.8
DBPC	87.3 ± 0.7	66.5 ± 1.0	69.6 ± 0.8	64.5 ± 1.2	62.9 ± 0.8	65.0 ± 0.9
DXPC	88.7 ± 1.1	59.6 ± 1.2	57.7 ± 1.5	53.5 ± 1.8	57.7 ± 1.4	69.7 ± 1.1

Supplementary Table S4: Stalk free energy values in kJ/mol with 1 SE for lipid bilayers with one polyL helix extracted from PMFs shown in Fig. 2B and Supplementary Fig. S8.

# aa in polyL	PIPC	DOPC	POPC	DPPC
10	0.2 ± 1.3	12.0 ± 1.1	21.8 ± 1.4	44.5 ± 0.9
12	4.9 ± 1.1	18.7 ± 1.2	26.7 ± 1.1	53.8 ± 1.2
14	9.0 ± 1.1	21.2 ± 1.0	30.1 ± 1.0	56.7 ± 1.1
16	12.0 ± 0.9	26.3 ± 0.9	35.8 ± 0.9	59.0 ± 0.9
18	15.5 ± 0.9	28.3 ± 0.9	40.0 ± 0.9	63.0 ± 0.8
20	19.3 ± 0.9	31.1 ± 1.0	45.2 ± 1.0	68.0 ± 0.7
22	19.3 ± 1.1	34.1 ± 1.1	46.7 ± 0.8	74.1 ± 0.8
24	22.3 ± 1.0	37.4 ± 0.7	50.7 ± 0.8	78.7 ± 0.5
26	22.2 ± 0.8	37.0 ± 0.9	52.4 ± 0.8	81.6 ± 0.8
28	21.7 ± 0.8	38.2 ± 0.8	51.1 ± 1.0	83.4 ± 0.6
30	22.5 ± 1.0	35.2 ± 1.0	50.6 ± 0.8	82.3 ± 0.6

Supplementary Table S5: Stalk free energy values in kJ/mol with 1 SE for POPC bilayers using Martini 3.0.0 with 1 to 4 TMDs extracted form PMFs shown in Fig. 1C and Supplementary Fig. S6.

#TMD	HA	RFVF-Gc	PrV-gB	syt	syb
1	46.0 ± 1.3	52.3 ± 1.0	50.6 ± 1.1	48.1 ± 1.6	51.5 ± 1.5
2	45.3 ± 1.2	51.1 ± 1.1	52.6 ± 1.1	47.9 ± 1.3	45.2 ± 1.3
3	35.0 ± 2.3	47.4 ± 1.2	45.4 ± 1.4	46.3 ± 1.2	40.3 ± 1.3
4	30.6 ± 2.4	38.9 ± 1.5	36.3 ± 1.6	38.6 ± 1.1	33.4 ± 1.2

Supplementary References

(1) Poojari, C. S.; Scherer, K. C.; Hub, J. S. Free energies of membrane stalk formation from a lipidomics perspective. *Nat. Commun.* **2021**, *12*, 1–10.

(2) Hub, J. S.; de Groot, B. L.; van der Spoel, D. g_wham–A Free Weighted Histogram Analysis Implementation Including Robust Error and Autocorrelation Estimates. *J. Chem. Theory Comput.* **2010**, *6*, 3713–3720.

(3) Smirnova, Y. G.; Risselada, H. J.; Müller, M. Thermodynamically reversible paths of the first fusion intermediate reveal an important role for membrane anchors of fusion proteins. *Proc. Natl. Acad. Sci. U.S.A.* **2019**, *116*, 2571–2576.

(4) Ollila, O. H. S.; Risselada, H. J.; Louhivuori, M.; Lindahl, E.; Vattulainen, I.; Marrink, S. J. 3D Pressure Field in Lipid Membranes and Membrane-Protein Complexes. *Phys. Rev. Lett.* **2009**, *102*, 078101.

(5) Vanegas, J. M.; Torres-Sánchez, A.; Arroyo, M. Importance of Force Decomposition for Local Stress Calculations in Biomembrane Molecular Simulations. *J. Chem. Theory Comput.* **2014**, *10*, 691–702.
